# Coronary Wave Intensity Analysis as an Invasive and Vessel-Specific Index of Myocardial Viability

**DOI:** 10.1161/CIRCINTERVENTIONS.122.012394

**Published:** 2022-12-20

**Authors:** Matthew Ryan, Kalpa De Silva, Holly Morgan, Kevin O’Gallagher, Ozan M. Demir, Haseeb Rahman, Howard Ellis, Luke Dancy, Daniel Sado, Julian Strange, Narbeh Melikian, Michael Marber, Ajay M. Shah, Amedeo Chiribiri, Divaka Perera

**Affiliations:** Cardiovascular Division, King’s College London, UK (M.R., K.D.S., H.M., K.O., O.M.D., H.R., H.E., M.M., A.M.S., D.P.).; Cardiology Department, King’s College Hospital, London, UK (L.D., D.S., N.M.).; Bristol Heart Institute, UK (J.S.).; Imaging Sciences Division, King’s College London, UK (A.C.).

**Keywords:** coronary physiology, coronary artery disease, heart failure, myocardial hibernation, reduced ejection fraction

## Abstract

Coronary angiography and viability testing are the cornerstones of diagnosing and managing ischemic cardiomyopathy. At present, no single test serves both needs. Coronary wave intensity analysis interrogates both contractility and microvascular physiology of the subtended myocardium and therefore has the potential to fulfil the goal of completely assessing coronary physiology and myocardial viability in a single procedure. We hypothesized that coronary wave intensity analysis measured during coronary angiography would predict viability with a similar accuracy to late-gadolinium–enhanced cardiac magnetic resonance imaging.

**Methods:**

Patients with a left ventricular ejection fraction ≤40% and extensive coronary disease were enrolled. Coronary wave intensity analysis was assessed during cardiac catheterization at rest, during adenosine-induced hyperemia, and during low-dose dobutamine stress using a dual pressure-Doppler sensing coronary guidewire. Scar burden was assessed with cardiac magnetic resonance imaging. Regional left ventricular function was assessed at baseline and 6-month follow-up after optimization of medical-therapy±revascularization, using transthoracic echocardiography. The primary outcome was myocardial viability, determined by the retrospective observation of functional recovery.

**Results:**

Forty participants underwent baseline physiology, cardiac magnetic resonance imaging, and echocardiography, and 30 had echocardiography at 6 months; 21/42 territories were viable on follow-up echocardiography. Resting backward compression wave energy was significantly greater in viable than in nonviable territories (−5240±3772 versus −1873±1605 W m^−2^ s^−1^, *P*<0.001), and had comparable accuracy to cardiac magnetic resonance imaging for predicting viability (area under the curve 0.812 versus 0.757, *P*=0.649); a threshold of −2500 W m^−2^ s^−1^ had 86% sensitivity and 76% specificity.

**Conclusions:**

Backward compression wave energy has accuracy similar to that of late-gadolinium–enhanced cardiac magnetic resonance imaging in the prediction of viability. Coronary wave intensity analysis has the potential to streamline the management of ischemic cardiomyopathy, in a manner analogous to the effect of fractional flow reserve on the management of stable angina.

**What is Known**

- Myocardial viability testing is an important diagnostic consideration in patients with ischemic cardiomyopathy.
- Coronary wave energy predicts functional recovery in patients with non–ST-segment–elevation myocardial infarction‚ but its use as a viability test in ischemic cardiomyopathy is unknown.

**What the Study Adds**

- Backward compression wave energy, measured with coronary wave intensity analysis, can be used to assess myocardial viability during cardiac catheterization.
- The accuracy of wave intensity analysis is similar to late-gadolinium–enhanced cardiac magnetic resonance imaging
- With further validation and technical development, coronary wave energy could be a useful index for routinely assessing viability in the catheterization laboratory.

Ischemic cardiomyopathy (ICM) accounts for 50% to 70% of cases of heart failure in North America and Europe.^1^ The prognosis of these patients remains poor, with mortality rates of approximately 50% at 5 years, often preceded by recurrent hospitalizations for decompensated heart failure.^2^ Improving left ventricular function is a key therapeutic goal in ICM, which is associated with improved survival and quality of life; optimal medical therapy, device therapy, and revascularization may all have roles in achieving such improvements, with recent data suggesting that optimal medical therapy is the primary driver of improved left ventricle function.^3,4^

Myocardial viability testing aims to prospectively identify regions of myocardium where function is likely to recover. At present, testing is achieved with noninvasive imaging methods, including late-gadolinium–enhanced cardiac magnetic resonance imaging (LGE-CMR), stress echocardiography, and nuclear imaging. Although well validated for the prediction of functional recovery, noninvasive viability testing has several limitations: it requires resources that are not universally available, certain modalities are frequently contraindicated in patients with ICM (such as CMR with cardiac devices), and the distribution of viability is not co-registered to the coronary anatomy. An analysis of viability testing in the STICH trial (Surgical Treatment for Ischemic Heart Failure) showed no incremental benefit to coronary artery bypass grafting in patients with extensive viability defined by dobutamine stress echocardiography or single photon emission computed tomography.^3^ Furthermore, the REVIVED-BCIS2 trial (Revascularization for Ischemic Ventricular Dysfunction) showed no benefit to viability-guided percutaneous coronary intervention (PCI), where most of the testing was performed with LGE-CMR and dobutamine stress echocardiography.^4^ A vessel-specific index of ischemia and viability may allow the identification of the substrate of hibernation and patients who may benefit from revascularization.^3^

At present, there is no established technique for assessing myocardial viability in a vessel-specific manner at the time of angiography. Coronary wave intensity analysis (cWIA) is a promising solution. cWIA uses simultaneous intracoronary pressure and Doppler flow data to measure the energy fluxes that drive or impede myocardial perfusion, characterized as forward (originating from the aorta) or backward (originating from the microcirculation) waves.^5^ We have previously demonstrated that the energy of the backward originating waves (backward expansion wave [BEW] and backward compression wave [BCW]) predict recovery of regional function in patients presenting with large non–ST-segment–elevation myocardial infarction.^6^

## Objectives

In the current study, we investigated whether cWIA could accurately assess myocardial viability in patients with stable ischemic cardiomyopathy treated with optimal medical therapy (with or without revascularization). Furthermore, we hypothesized that cWIA derived during dobutamine stress would improve the prediction of viability compared with measurements in resting conditions.

## Methods

The data that support the findings of this study are available from the corresponding author upon reasonable request. Patients with a recent diagnosis of ICM who were scheduled for coronary angiography and/or percutaneous coronary intervention at one of the following UK centers were enrolled in the study: St Thomas’ Hospital, London; King’s College Hospital, London; Bristol Heart Institute, Bristol. Inclusion criteria were impaired left ventricular systolic function (ejection fraction ≤40%) and extensive coronary artery disease (British Cardiovascular Intervention Society Jeopardy Score ≥8).^7^ Exclusion criteria were acute coronary syndrome within 4 weeks, contraindication to LGE-CMR, and severe valvular heart disease. All participants gave written informed consent in accordance with the protocol approved by the UK National Research Ethics Service (18/LO/0171). The study was registered with the National Institute for Health Research UK Clinical Research Network Portfolio database (Central Portfolio Management System identifier: 36867).

### Catheterization Protocol

Cardiac catheterization was performed using 6Fr catheters from either the radial or femoral arteries. Intracoronary isosorbide dinitrate (1 mg for the left coronary, 600 mcg for the right coronary) was administered. After assessment of the coronary anatomy, a dual pressure-Doppler flow sensing 0.014” coronary guidewire (Combowire, Philips Volcano, CA) was used to measure distal coronary pressure and instantaneous peak flow velocity, as previously described.^8^ Aortic pressure was measured from the guide catheter. Measurements were performed in all vessels with a visually significant stenosis unless the operator felt that a pressure wire study could not be safely performed without disrupting the lesion or if the vessel was occluded. Hemodynamic measurements were recorded under resting conditions, during intracoronary adenosine-mediated hyperemia (up to 120 mcg in the left coronary and 96 mcg in the right coronary) and at the end of each stage of an incremental low-dose dobutamine infusion (5 mcg kg min^−^^1^ for 3 minutes, and then 10 mcg kg min^−^^1^ for 3 minutes). The myocardial segments subtended by each diseased vessel was determined using a prespecified algorithm based on the American Heart Association 17 segment model, by 2 independent observers who were blinded to the physiological, echocardiographic, and CMR data (Supplemental Materials: segmental attribution tool).^9^ This segmental attribution was used to determine regional viability. Angiographic stenosis severity was measured by quantitative coronary angiography.

### Physiological Data Processing

Pressure and flow data were exported and analyzed using custom-made software (StudyManager, Academic Medical Centre, University of Amsterdam, the Netherlands and Cardiac Waves, King’s College London, UK). Fractional flow reserve (FFR) and coronary flow reserve (CFR) were determined using pan-cycle pressure and flow data (FFR=P_d_/P_a_ during hyperemia, CFR=average peak velocity (APV)_hyperemia_/APV_rest_). Wave intensity was calculated as the product of the derivatives of distal coronary pressure (P) and flow velocity (U), (dP/dt × dU/dt), and wave separation performed as previously described (Figure S1).^10,11^

### Cardiac MRI

Scans were preferentially performed at 3-Tesla (T) (Achieva, Philips Healthcare, Amsterdam, the Netherlands). Where this was not possible (claustrophobia, high body mass index), a 1.5 T scanner was used (Aera, Siemens Healthcare, Erlangen, Germany). The same protocol was used on the 2 scanning platforms, with a dual-RF transmission and a 32-channel phased array body coil. LGE images were acquired at least 10 minutes after the administration of a 0.15 mmol/kg bolus of gadobutrol. Scar quantification was performed using the PSIR-LGE images in the short-axis; voxels with a signal intensity 5 standard deviations greater than normal myocardium were identified as scar. The standard clinical threshold of ≤25% LGE across the subtended territory was used to predict viability.

### Echocardiography, Medical Therapy, and Revascularization

All 2-dimensional transthoracic echocardiography studies were performed according to the British Society for Echocardiography standard protocol.^12^ Wall motion was scored qualitatively using a 5-point standard scale (1: normal, 2: mildly hypokinetic, 3: severely hypokinetic, 4: akinetic, 5: dyskinetic) and averaged across the territory subtended by each vessel, as above. The primary end point was myocardial viability, defined by a ≥0.5 reduction in average wall motion score between baseline and 6-month follow-up. All patients received maximally tolerated optimal medical therapy: the quality of optimal medical therapy was assessed at baseline and at follow-up (Table S2). Revascularization was performed with PCI, as clinically indicated or as per randomization in the REVIVED-BCIS2 trial,^13^ either during the same procedure or at a subsequent procedure following multidisciplinary discussion.

### Statistical Analysis

Statistical analyses were performed using SPSS, version 27. After visually assessing for the normality of data, continuous and normally distributed data were reported as mean±standard deviation and compared using paired (within groups) and unpaired (between groups) *t*-tests. Nonnormally distributed continuous data were reported as median [first quartile–third quartile] and compared using the Mann-Whitney-U and Wilcoxon signed rank tests as appropriate. All analyses were performed in a 2-tailed manner, with a *P* value <0.05 taken to indicate statistical significance. The ability of each wave to identify viability was assessed by receiver operator characteristic curve analysis and compared with the predictive accuracy of LGE-CMR using the DeLong method^14^ with optimum sensitivity, specificity, positive predictive value, and negative predictive value determined by Youden index.^15^ All authors had access to the data and take responsibility for its integrity and analysis.

### Precision Calculation

In the previously described acute coronary syndrome study, a BEW threshold of 2800 W m^−^^2^ s^−^^1^ had a sensitivity of 0.91 for predicting viability.^6^ Assuming a 75% rate of the primary end point, we calculated that a sample size of 40 territories would give an absolute precision of 0.1 for the 95% CIs for the same point estimate of 0.91.^16^

## Results

**Figure 1.**
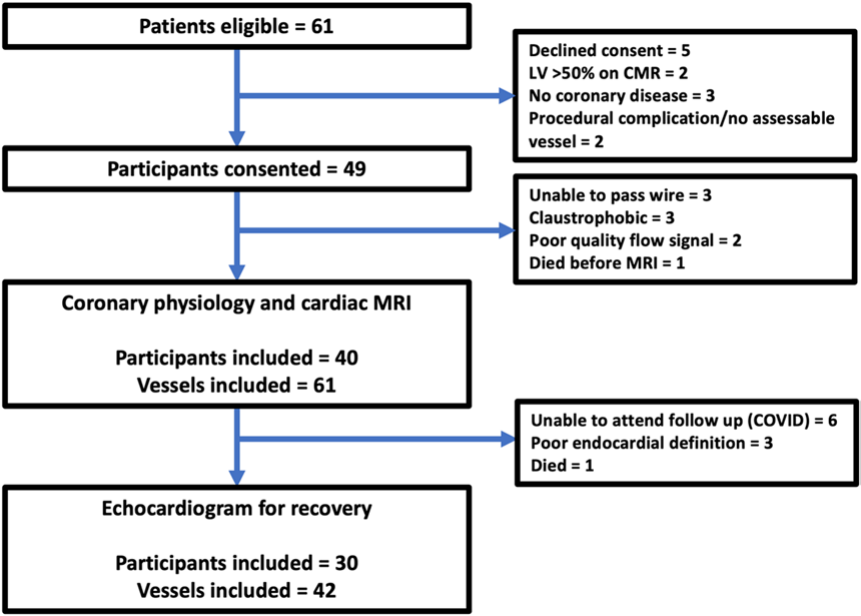
**Consort diagram.** CMR indicates cardiac magnetic resonance imaging; LV, left ventricle; and MRI, magnetic resonance imaging.

Forty participants were recruited (Figure 1). Sixty-one vessels were studied (left anterior descending artery in 34, circumflex artery in 15, and right coronary artery in 12). The mean number of myocardial segments subtended per vessel was 4 [3–5]. Median angiographic stenosis severity by quantitative coronary angiography was 72% [55–84]. Baseline echocardiography and LGE-CMR were performed in all participants. Eighteen participants were co-enrolled in the REVIVED-BCIS2 trial.^4^

Thirty participants had follow-up echocardiography after a median of 223 [175–346] days accounting for 42 vascular territories; 29 of these territories were revascularized with PCI, the remaining 13 were not revascularized. The quality of medical therapy improved in 14 participants (22 territories). The follow-up population did not differ significantly from the population who underwent baseline testing (Table 1).

**Table 1.**
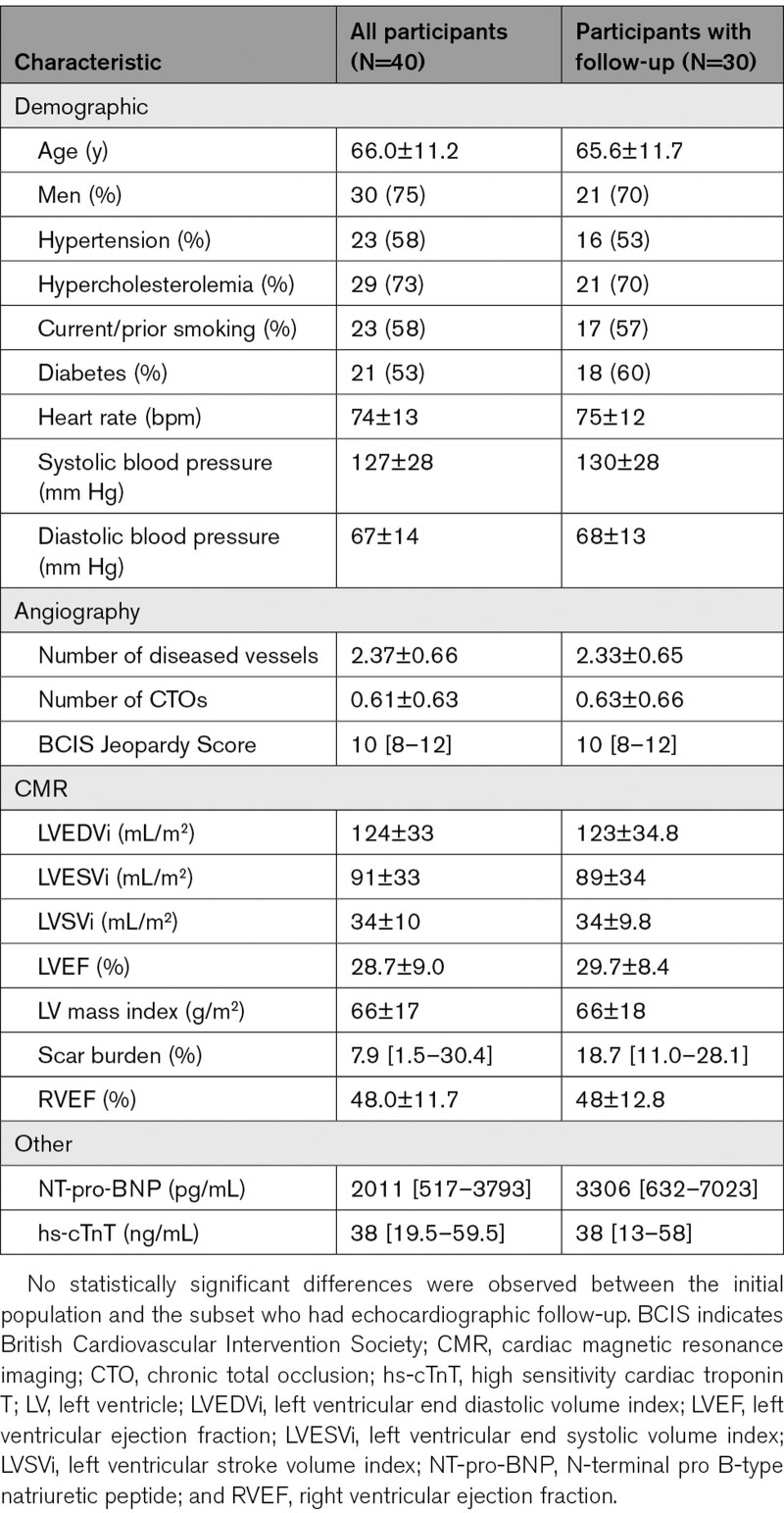
Participant Characteristics

### cWIA and Viability

Myocardial viability was observed in 21/42 territories (50%); the change in WMSI was −0.75 [−0.55 to −1.13] in the viable territories versus 0 [0 to 0.33] in the nonviable territories (*P*<0.001). There was no significant difference between viable and nonviable territories in the number of segments subtended (4 [4–5] versus 4 [3–6], *P*=0.876) or baseline wall motion (WMSI 3.14±0.45 versus 2.94±0.62, *P*=0.099). Viability at follow-up was similar in revascularized and non-revascularized territories (15/29 versus 6/13, *P*=0.739).

**Figure 2.**
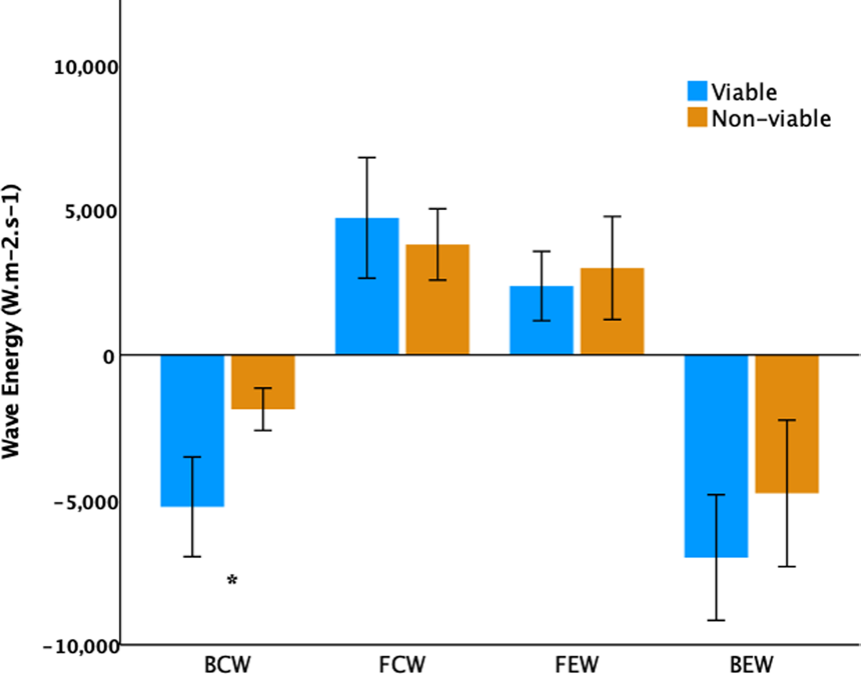
**Comparison of resting wave energy between viable and nonviable myocardium.** Error bars indicate the 95% CI. Asterisk indicates statistically significant difference (*P*<0.05). BCW indicates backward compression wave; BEW‚ backward expansion wave; FCW‚ forward compression wave; and FEW‚ forward expansion wave.

**Figure 3.**
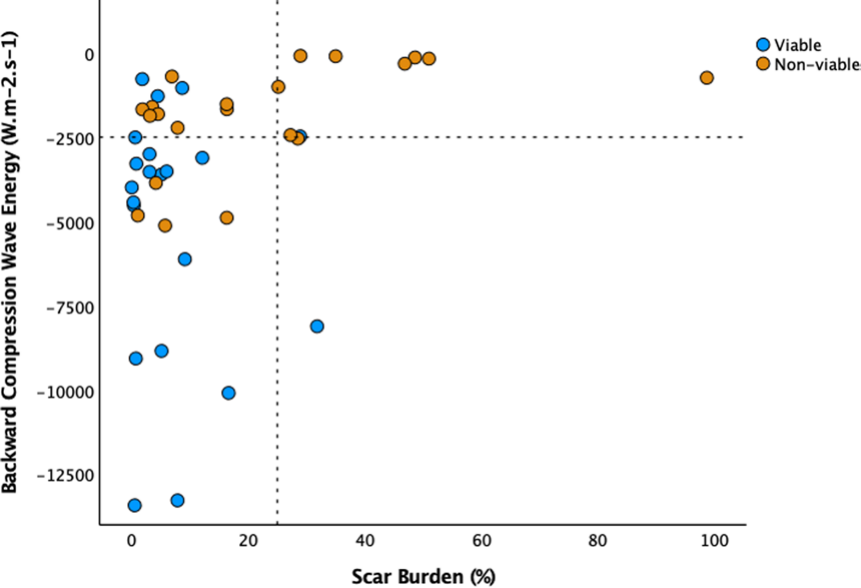
**Scatterplot comparing resting backward compression wave energy with scar burden in viable and nonviable territories.** The horizontal dotted line indicates the backward compression wave threshold derived from this dataset, whereas the vertical dotted line indicates the standard clinical viability threshold of ≤25% scar burden.

The resting BCW was significantly larger in viable territories (−5240±3772 versus −1873±1605 W m^−2^ s^−1^, *P*<0.001), but there was no significant difference in the magnitude of the BEW or the forward waves (Figures 2 and 3).

**Figure 4.**
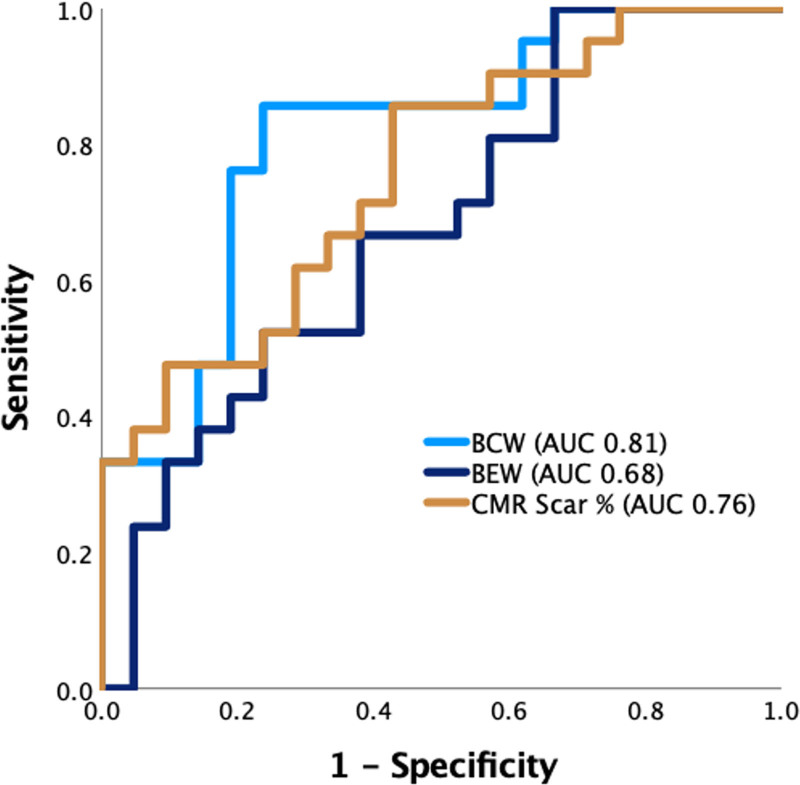
**Receiver operator characteristic curve for the prediction of viability.** Comparison between resting backward compression wave (BCW)‚ resting backward expansion wave (BEW)‚ and regional scar burden assessed with late-gadolinium–enhanced cardiac magnetic resonance imaging (LGE-CMR). For all indices, lower/more negative values indicate a more positive test. AUC indicates area under the curve.

The resting BCW had good overall accuracy for the prediction of viability (area under the curve, 0.812, [95% CI, 0.680–0.944]; *P*=0.001; Figure 4). The optimal BCW threshold was −2500 W m^−2^ s^−1^, which provided a sensitivity of 0.86, specificity of 0.76, positive predictive value of 0.78, and negative predictive value of 0.84. The BEW also predicted viability, but with lower accuracy (area under the curve, 0.680 [0.518–0.843]; *P*=0.046).

LGE-CMR–derived scar percentage was lower in viable than in nonviable territories (4.5% [0.6–8.9] versus 16.3% [4.3–32.0], *P*=0.004). Of the 42 territories in which remodeling was assessed, 31 territories had ≤25% scar burden. Viability was observed more frequently in territories with ≤25% scar burden (19/31 versus 2/11, *P*=0.014). LGE-CMR was an effective predictor of viability on receiver operator characteristic analysis (area under the curve, 0.757 [0.614–0.901]; *P*=0.004). The optimal cut-off was a scar burden of 12.1%, which provided a sensitivity of 0.86, specificity of 0.57, positive predictive value of 0.67, and negative predictive value of 0.80. The area under the curve for scar percentage was comparable to BCW (*P*=0.588; Figure 4).

### Fractional Flow Reserve, Coronary Flow Reserve, and Logistic Regression Analysis

FFR was numerically lower in nonviable territories; however, the relationship did not reach statistical significance (0.81±0.17 versus 0.71±0.16, *P*=0.058). CFR was similar between viable and nonviable territories (1.84±0.83 versus 2.20±1.02, *P*=0.423). Multivariate logistic regression analysis demonstrated that resting BCW magnitude and improvement of medical therapy were the only independent predictors of functional recovery, whereas BEW, FFR, revascularization, and wall motion score at baseline were not predictive (Table 2).

**Table 2.**
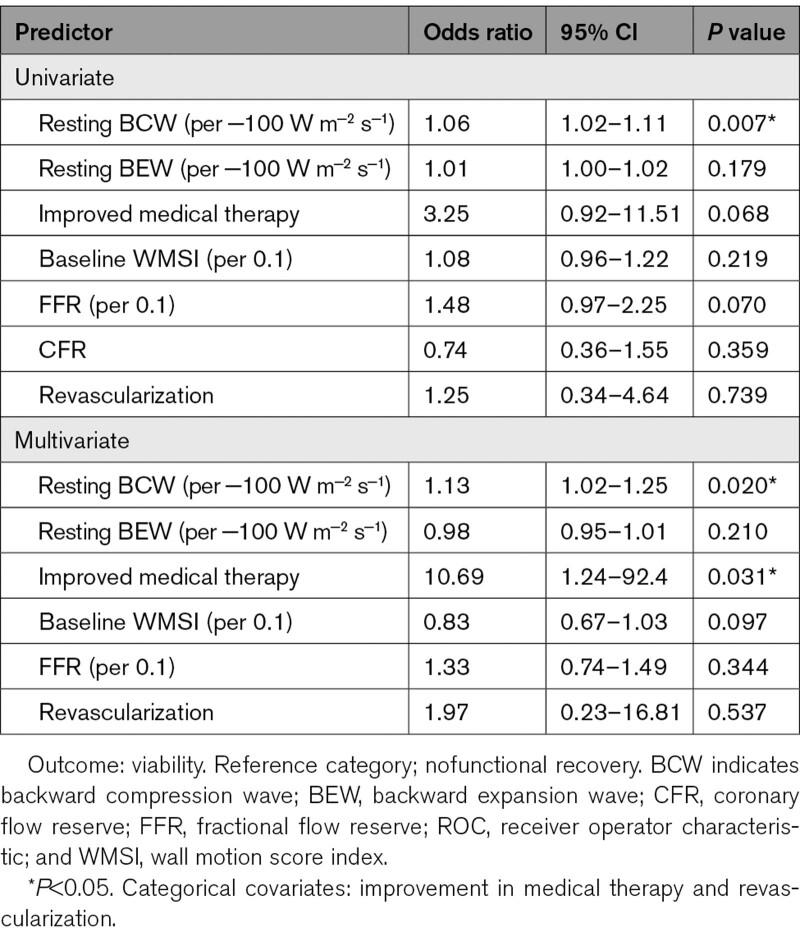
Univariate and Multivariate Logistic Regression for the Prediction of Functional Recovery From Physiological Parameters Identified From ROC Analysis and Controlled for the Presence of Revascularization and Improvement in Medical Therapy

### Dobutamine Testing

Details of the hemodynamic effects of dobutamine across the whole study population are included in the Supplemental Materials (Table S1 and Figure S2).

In 33 territories assessed with dobutamine where follow-up echocardiography data were available, there was a strong trend toward higher wave BCW energy in viable territories but no significant difference in other wave energies, whether expressed as absolute values during dobutamine administration or as changes in wave energy from resting conditions. BCW energy during dobutamine stress was predictive of viability, with accuracy comparable to assessment at rest (Figure S3).

## Discussion

We have demonstrated for the first time that resting BCW energy, derived from invasive measurements taken during cardiac catheterization, is maintained in viable myocardium and attenuated in nonviable myocardium. Consequently, BCW energy predicts viability, assessed by regional left ventricle functional recovery, with accuracy comparable to LGE-CMR. Although cWIA during low dose dobutamine stress was also predictive, its use did not improve diagnostic performance, indicating that a simpler assessment during resting conditions is sufficient.

### Backward Wave Energy

Wave intensity analysis was first applied to the coronary circulation 20 years ago.^17^ Since that time, cWIA has served as a useful research tool, facilitating greater understanding of the mechanisms of inducible ischemia and microvascular function. Avenues for translation into a clinical application have, however, not been forthcoming.

Previous work identified that the BEW was the best predictor of functional recovery in patients with recent non–ST-segment–elevation myocardial infarction‚ which contrasts with the results of our study in patients with chronic coronary syndromes.^6^ While the mechanisms generating each coronary wave remain imperfectly understood, the BEW is generated by isovolumic relaxation and its magnitude relates to subendocardial perfusion and microvascular function, both of which are more perturbed where there is an acute injury to the myocardium. Conversely, the BCW is generated by compression of the microcirculation during isovolumic contraction in early systole; it is thought to be inotropy-dependent and related to cardiac contractility, with significant increases in BCW energy observed with exercise.^18^ The larger BCW observed in viable myocardium may therefore reflect subclinical differences in contractility relating to preserved functional cardiomyocytes or higher adrenoceptor density in these territories.

### Fractional Flow Reserve

A low FFR did not predict viability in our study. Prior studies, which used single photon emission computed tomography to define viability, indicated an inverse correlation between FFR values and the mass of viable myocardium subtended^19^ and lower FFR values were associated with a higher chance of functional recovery compared with nonviable territories with an equivalent angiographic stenosis severity.^20^ The trend toward lower FFR values in nonviable territories in our study likely relates to higher stenosis severity in these regions. As stenosis resistance remains the key determinant of FFR values, and as vessels with greater disease burden are more likely to have sustained previous infarction, these results highlight that while in theory a given stenosis should have a lower FFR value in a viable than a nonviable territory, in clinical practice it is impossible to determine what FFR “should” be, and therefore extrapolate a “difference” related to the presence or absence of viability.

### Relationship Between Viability and Clinical Outcomes

In the recently published REVIVED-BCIS2 randomized trial, viability-guided PCI did not improve outcomes in patients with ICM, compared with optimal medical therapy alone.^4^ Although there was a modest increase in left ventricular ejection fraction in both arms of the trial, PCI provided no incremental improvement in left ventricular function.

Although viability-guided PCI does not provide prognostic benefit to patients with ICM, this should not be taken to mean that viability testing itself is redundant. The relationship between myocardial viability, functional recovery, and clinical outcomes needs to be more clearly defined; these relations are relevant not only to revascularization but to medical and device therapy, as well as predicting prognosis. Viability may also be considered when selecting coronary territories for revascularization with ICM and limiting angina (a population were excluded from REVIVED and still warrant revascularization on symptomatic grounds).

### Limitations

Although adequately powered for the prespecified primary end point, the study has a relatively small sample size and experienced significant loss to follow-up because of the COVID-19 pandemic, although analysis of demographic features suggest no systematic difference in those patients who were lost to follow-up. Physiological assessment was not possible in around 10% of vessels attempted, due to challenging anatomy or procedural factors. Furthermore, this method cannot be used to assess the viability of territories subtended by chronic total occlusions or vessels with critical stenoses, a subset of coronary lesions to which viability testing is relevant. However, these limitations would not prevent the adoption of invasive assessment as part of a wider armamentarium of testing strategies, with multiple modalities available for different clinical circumstances.

The present study included few territories with extensive infarction, with 90% having <50% scar burden. This likely represents a degree of selection bias in a population who were being considered for revascularization, a consistent issue identified in prior studies and clinical trials including STICH and PARR-2.^21,22^ External validation of these findings in a separate cohort of patients would be a valuable next step.

### Future Directions for Clinical Application of cWIA

The acquisition and processing of cWIA are currently limited to academic institutions with specific expertise. Through our multi-center design, we have demonstrated the wider feasibility of this technique, though the general trend in coronary physiology is toward simpler streamlined clinical tools. Increasing interest in the coronary microvasculature, as well as the development of clinical utilities for cWIA as demonstrated in this study, shows the potential for Doppler-based assessment to accurately and efficiently identify patients who would benefit from individualized, physiology-guided therapies, and may stimulate development. Following echocardiography, patients might undergo a full assessment of etiology, coronary disease severity, and viability in a single procedure; a factor that may be of particular benefit to those in whom other imaging is contraindicated. Finally, cWIA demonstrates the potential to separate patients with low scar burden by LGE-CMR, where specificity for viability is low, likely related to the presence of longstanding hibernation, which is irreversible despite the absence of infarction.

## Conclusions

Backward compression wave energy measured with cWIA accurately predicts myocardial viability at medium-term follow-up in patients with ischemic cardiomyopathy. Invasive coronary physiology may therefore provide a useful adjunct to diagnostic angiography in the assessment of patients with suspected ischemic cardiomyopathy. The administration of dobutamine does not increase the accuracy of invasive assessment in predicting viability.

## Article Information

### Sources of Funding

This work was supported by the British Heart Foundation through a clinical research training fellowship award (FS/18/16/33396). The authors are further supported through grants from the British Heart Foundation (FS/16/49/32320, FS/CRTF/21/24190, PG/19/9/34228, CH/1999001/11735, RE/18/2/34213, RM/17/3/33381) and from the National Institute for Health Research (NIHR130593 and 10/57/67). From the British Heart Foundation Centre of Excellence at the School of Cardiovascular and Metabolic Medicine and Sciences, King’s College London, London, United Kingdom and the School of Biomedical Engineering and Imaging Sciences, King’s College London, London, United Kingdom, and the National Institute for Health Research via the Biomedical Research Centre award to Guy’s and St. Thomas’ Hospital and King’s College London.

### Disclosures

### None.

### Supplemental Material

Figures S1–S3

Tables S1–S2

## Supplementary Material


