# Supplementary material for: Coronary Wave Intensity Analysis as an Invasive and Vessel-Specific Index of Myocardial Viability
Source: Circ Cardiovasc Interv. 2022 Dec 20;15(12):e012394. doi: 10.1161/CIRCINTERVENTIONS.122.012394 (PMC9760472; doi:10.1161/CIRCINTERVENTIONS.122.012394)
Supplement: Supplementary file 1 [file hcv-15-e012394-s001.pdf]

Supplementary Methods - Segmental Attribution Tool

Wire location	Anatomy	Segments	Additions
Proximal LAD	Proximal to 1st diagonal	1,2,7,8,13,14	15 if wrap-around
Mid LAD	Beyond 1st diagonal	7,8,13,14	15 if wrap-around
Distal LAD	Beyond 2nd diagonal	13,14	15 if wrap-around
First diagonal	If higher than OM1 and no IM	1,7,13	
	If lower than OM1 or IM	7, 13	
Proximal LCx (above OM1)	Dominant (4 branches including PDA)	3,4,5,6,9,10,11,12,15,16	
	Co-dominant (3 OM branches, no PDA)	4,5,6,10,11,12,16	
	2 OM branches	5,6,11,12	16 if large/reaches apex
	1 OM branch	6,12	16 if large/reaches apex
Mid LCx (beyond OM1)	Dominant (4 branches including PDA)	3,4,5,9,10,11,15,16	
	Co-dominant (3 OM, branches, no PDA)	4,5,10,11,16	
	2 OM branches	5,11,	16 if large/reaches apex
	1 OM branch	N/A	
Distal LCx (beyond OM2)	Dominant (4 branches including PDA)	3,4,9,10,15	
	Co-dominant (3 OM, branches, no PDA)	4,10,16	
	2 OM branches	N/A	
	1 OM branch	N/A	
First OM		6,12	16 if largest OM/reaches apex
Second obtuse marginal		5,11	16 if largest OM/reaches apex
Third obtuse marginal		4,10	16 if largest OM/reaches apex

Intermediate	If D1 higher than OM1	6,12	16 if largest lateral wall vessel/reaches apex
	If OM1 higher than D1	1,7	16 if largest lateral wall vessel/reaches apex
Left Main	Combine segments for proximal LAD/LCx		
RCA (before crux)	Dominant with 3 PLV branches	3,4,5,6,9,10, 11,12,15,16	Remove 15 is wrap around LAD
	Dominant with 2 PLV branches	3,4,5,9,10,1 1,15	Remove 15 is wrap around LAD
	Dominant with 1 PLV branch	3,4,9,10,15	Remove 15 is wrap around LAD
	Dominant with no PLV (PDA only)	3,9,15	Remove 15 is wrap around LAD
	Non-dominant	N/A	
Beyond crux in PLV system	3 PLV branches	4,5,6,10,11, 12,15	Remove 15 is wrap around LAD
	2 PLV branches	4,5,10,11	15 if large/reaches apex
	1 PLV branch	4,10	15 if large/reaches apex
First PLV		4,10	16 is largest PLV/reaches apex
Second PLV		5,11	16 is largest PLV/reaches apex
Third PLV		6,12	16 is largest PLV/reaches apex
PDA	Proximal PDA	3,9,10,15	Remove 15 is wrap around LAD
	Distal PDA	9,10,15	Remove 15 is wrap around LAD

Guidance Notes

1. The stenosis location is taken to be the most distal angiographically significant stenosis (>50%).
2. Side branches are considered significant if more than 2mm in diameter. This applies for both defining the proximal/mid/distal segments, and for determining the number of lateral wall branches.

3. Where more than one lateral wall branch (>2mm) arises from a common trunk, rather than the main epicardial vessel, these should be considered to be successive branches, for example:
    a. a single large OM1 arises from the AV circumflex, then bifurcates into two 2mm daughter vessels, these should be considered OM1 and OM2

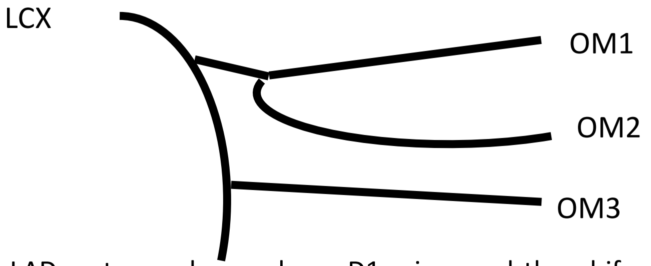

    b. a "dual" LAD system, where a large D1 arises and then bifurcates into three 2mm daughter vessels, should be considered D1, D2, D3.

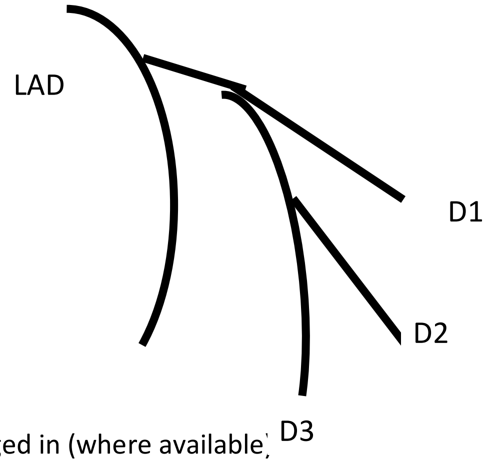

4. Additional segments best judged in (where available)
    a. Wrap around LAD – RAO caudal
    b. OM/PLV – LAO cranial
5. If there is uncertainty in regard to the distribution of lateral wall vessels the left coronary angiogram should be reviewed in the LAO caudal view and related to the AHA segmentation, with the position of the proximal LAD aligned on the junction of segments 1 and 2. For example, if there appear to be both a large OM2 and large PLV2, then the LCx anatomy is reviewed to determine whether the circumflex vessels reach the region of segment 5 and 11.

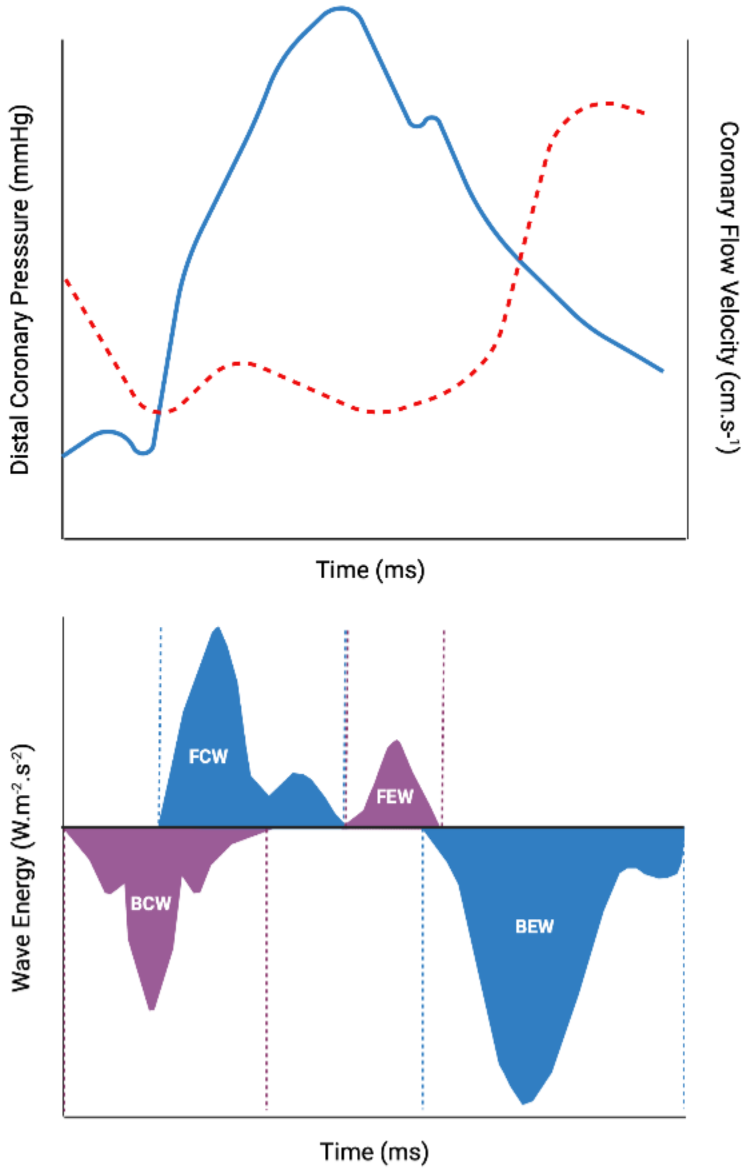

Figure S1 – Diagram of a typical coronary wave intensity analysis profile. Top panel: blue line indicates distal coronary pressure, red line indicates coronary flow velocity. Bottom panel: forward (aortic) originating waves have a positive vector and are displayed above the line; backward (myocardial) originating waves have a negative vector and are displayed below the line. Blue color indicates accelerating waves, purple color indicates decelerating waves. Dotted lines demarcate the start and end of each wave. Pressure and flow

waveforms were ensemble averaged over five cardiac cycles, before being smoothed with a Savitsky-Golay filter. Wave speed was calculated using the single-point method for the resting condition and then fixed at this speed during pharmacological stress in accordance with the prior observation that wave speed does not change during conditions of hyperaemia.

Four major waves were identified. The backward compression wave (BCW) is generated by myocardial compression during isovolumic contraction in early systole and is responsible for slowing of coronary blood flow, which remains reduced throughout systole. The BCW is identified as the largest backward originating and decelerating wave arising after the start of the cardiac cycle. The forward compression wave (FCW) is generated by rising aortic pressure after aortic valve opening in late systole and is identified as the first forward originating and accelerating wave arising after the BCW. The forward expansion wave (FEW) is generated by falling aortic pressure just prior to and following aortic valve closure and is identified as the largest forward originating and decelerating wave arising after the FCW. The backward expansion wave (BEW) is generated by isovolumic relaxation and is responsible for the rapid acceleration of coronary flow in early diastole which is a key driver of coronary perfusion. The BEW is identified as the largest backward-originating and accelerating wave arising after the end of the BCW and FCW. Wave energy is determined from the area under the curve of each wave – forward originating waves are expressed as positive values and backward originating waves are expressed as negative values.

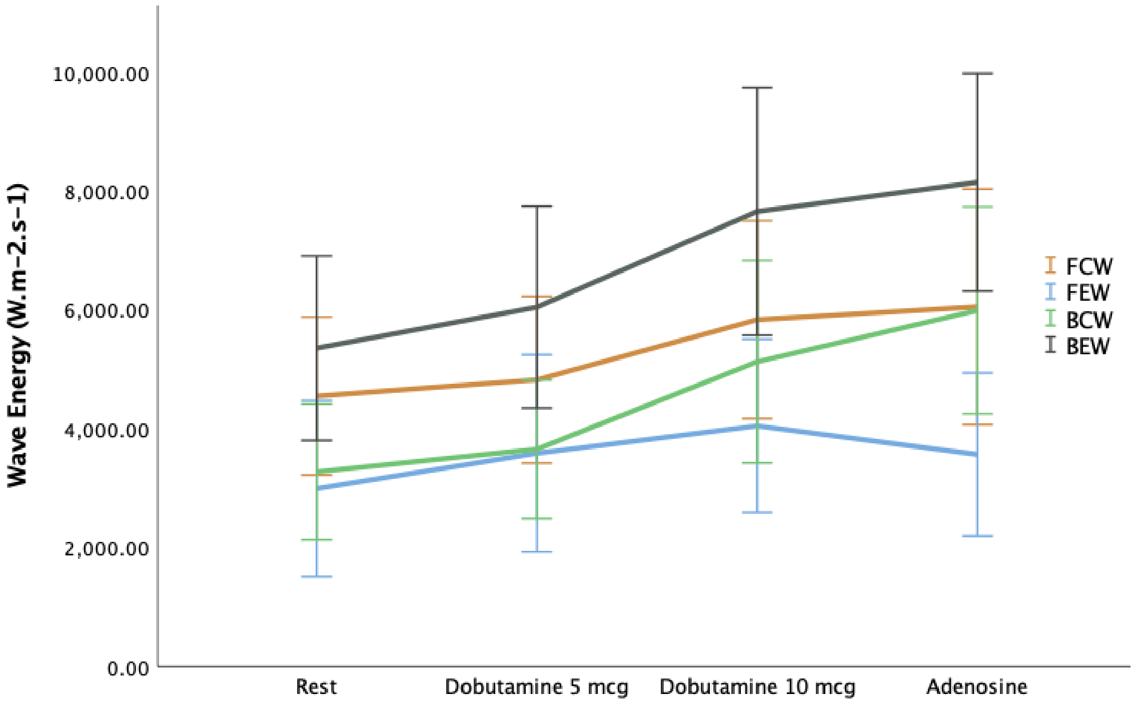

Figure S2 – Magnitude of coronary wave energy during dobutamine administration. Coronary wave energy was altered by dobutamine administration (total wave energy 16169 ± 11442 vs. 22657 ± 14383 W.m-2.s-1, $p < 0.001$), with the energy of the forward waves increasing at 5 mcg.kg-1.min-1, and all waves having significantly increased in magnitude at 10 mcg.kg-1.min-1; the backward wave energies had greater increases than the forward waves between 5 mcg.kg-1.min-1 and 10 mcg.kg-1.min-1. Error bars indicate 95% confidence intervals.

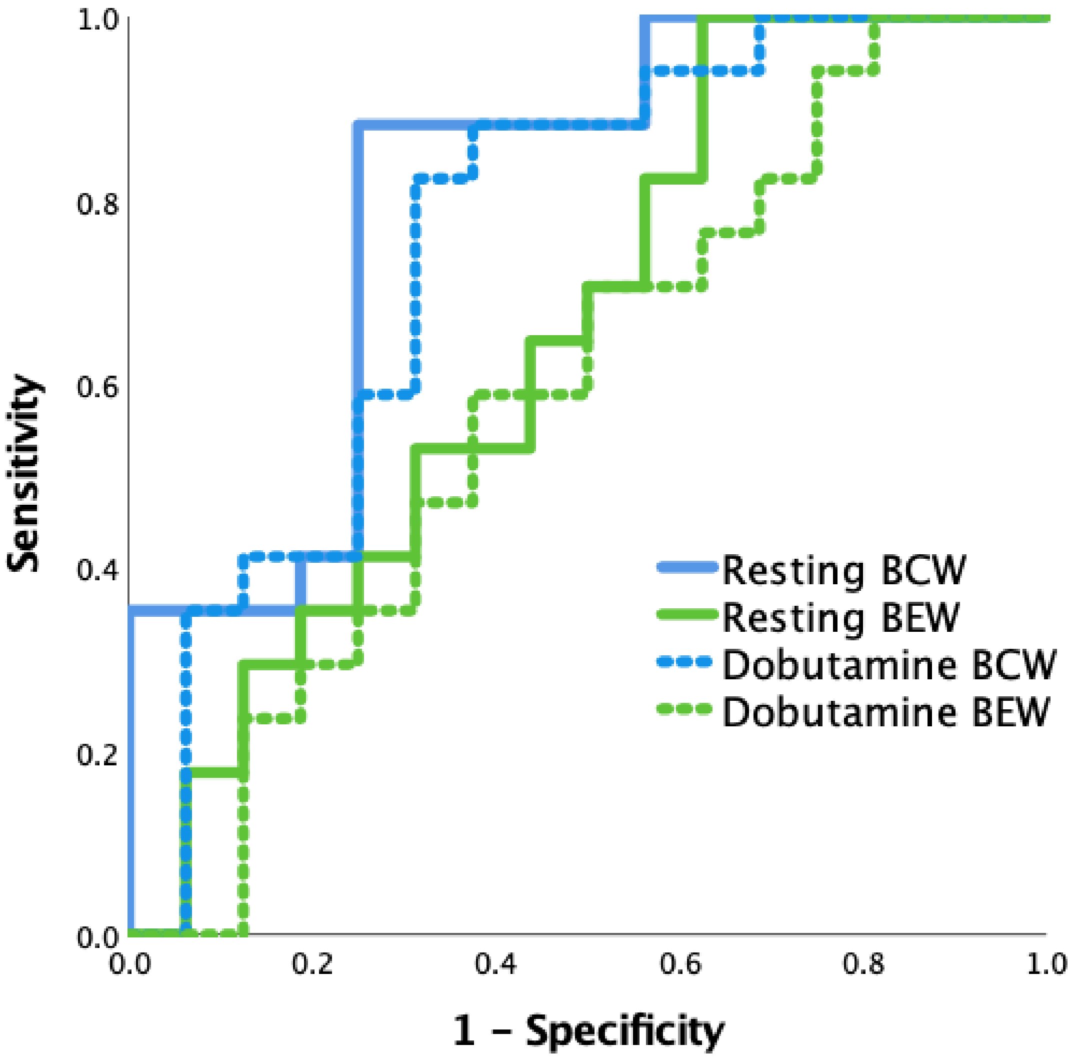

	Area	95% CI	p-value
Resting BCW	0.805	0.651-0.959	0.003
Resting BEW	0.654	0.463-0.846	0.130
Dobutamine BCW	0.757	0.586-0.929	0.012
Dobutamine BEW	0.592	0.393-0.791	0.368

Figure S3 – ROC curve for the prediction of viability. Comparison between assessment in resting conditions (solid line) and during dobutamine stress (dotted line). Area under the curve are displayed below the curve.

**Optimal Medical Therapy – Drugs and Devices**	RAAS: those affecting the renin/angiotensin/aldosterone system (angiotensin converting enzyme inhibitors (ACEi), angiotensin receptor blockers, angiotensin receptor/neprilysin inhibitors (ARNI))<br><br>Beta-blockers<br><br>Mineralocorticoid receptor antagonists<br><br>cardiac resynchronization therapy pacemaker (CRT-P)
**Definition of Improvement in OMT**	Two or more of:<br><br>Addition of another drug class<br><br>*and/or*<br><br>Increase in drug dose<br><br>*and/or*<br><br>Change to a more potent drug within the class (such as ACEi to ARNI)<br><br>*and/or*<br><br>Implantation of a CRT-P

Table S1 – Definition of OMT and Improvement in Medical Therapy

Condition	Resting	Dobutamine 5 mcg.kg^{-1}.min^{-1}	Dobutamine 10 mcg.kg^{-1}.min^{-1}
**Heart rate (bpm)**	75.8 ± 12.9	75.9 ± 13.2	**80.6 ± 14.7***
**Mean arterial pressure (mmHg)**	90.9 ± 17.7	90.4 ± 15.1	91.3 ± 17.1
**P_d/P_a**	0.81 ± 0.19	0.81 ± 0.19	**0.80 ± 0.20***
**Average peak velocity (cm.s^{-1})**	21.2 ± 10.5	21.3 ± 9.6	22.0 ± 9.9
**Microvascular resistance (mmHg.s.cm^{-1})**	4.64 ± 3.23	**4.33 ± 2.82***	**4.03 ± 2.30***
**Rate-pressure product (mmHg.bpm)**	9450 ± 2749	9564 ± 2408	**10536 ± 3089***

Table S2 - Effects of dobutamine on pan-cycle coronary hemodynamics and coronary wave energy. All conditions compared with paired T-Tests to resting condition. Asterisk and bold font indicate a statistically significant difference ($p < 0.05$).
